# Contemporary Radical Prostatectomy

**DOI:** 10.1155/2011/645030

**Published:** 2011-04-14

**Authors:** Qiang Fu, Judd W. Moul, Leon Sun

**Affiliations:** Division of Urology, Department of Surgery, Duke Prostate Center, Duke University Medical Center, P.O. Box 3707, Durham, NC 27710, USA

## Abstract

*Purpose*. Patients diagnosed with clinically localized prostate cancer have more surgical treatment options than in the past. This paper focuses on the procedures' oncological or functional outcomes and perioperative morbidities of radical retropubic prostatectomy, radical perineal prostatectomy, and robotic-assisted laparoscopic radical prostatectomy. *Materials and Methods*. A MEDLINE/PubMed search of the literature on radical prostatectomy and other new management options was performed. *Results*. Compared to the open procedures, robotic-assisted radical prostatectomy has no confirmed significant difference in most literatures besides less blood loss and blood transfusion. Nerve sparing is a safe means of preserving potency on well-selected patients undergoing radical prostatectomy. Positive surgical margin rates of radical prostatectomy affect the recurrence and survival of prostate cancer. The urinary and sexual function outcomes have been vastly improved. Neoadjuvant treatment only affects the rate of positive surgical margin. Adjuvant therapy can delay and reduce the risk of recurrence and improve the survival of the high risk prostate cancer. *Conclusions*. For the majority of patients with organ-confined prostate cancer, radical prostatectomy remains a most effective approach. Radical perineal prostatectomy remains a viable approach for patients with morbid obesity, prior pelvic surgery, or prior pelvic radiation. Robot-assisted laparoscopic prostatectomy (RALP) has become popular among surgeons but has not yet become the firmly established standard of care. Long-term data have confirmed the efficacy of radical retropubic prostatectomy with disease control rates and cancer-specific survival rates.

## 1. Introduction

With regard to oncological outcomes and perioperative morbidity of prostate cancer, radical prostatectomy (RP) of localized prostate cancer has dramatically improved the results of surgical treatment [1]. It has two approaches: open and minimally invasive prostatectomy. Open prostatectomy includes radical retropubic (RRP) and perineal prostatectomy (RPP). Laparoscopic radical prostatectomy (LRP) and robotic-assisted laparoscopic radical prostatectomy (RALP) comprise minimally invasive prostatectomy. Compared to the open procedures, minimally invasive radical prostatectomy has increased in recent years [2]. RALP can achieve excellent oncological and functional outcomes. Pure LRP has a steep learning curve, but RALP is easier to learn and is now the surgical treatment of choice in most centers of excellence in the United States. In 2007, it was estimated that approximately 63% of RPs for localized prostate cancer were performed using the robot-assisted approach, 36% by an open approach, and less than 1% by the pure laparoscopic technique [3, 4]. There is now a significant body of literature comparing prostate cancer outcomes after RRP, LRP, and RALP [5–7]. This paper focuses on the procedures' oncological or functional outcomes and perioperative morbidities of RRP, RPP, and RALP.

## 2. Types of Radical Prostatectomy

In 1945, Millin described, for the first time, the transabdominal retroperitoneal approach to facilitate operation on the prostate. This approach was used to perform simple prostatectomies [8], and it was not until 1949 that Memmelaar used the same approach to perform RRP for the first time [9]. Initially, the procedure was accompied with a high incidence of complications, namely total impotence, incontinence, and excessive hemorrhage. Over the last few decades, improved delineation of the surgical anatomy (including the dorsal venous complex [10] and the anatomic relevance of the striated sphincter [11]) enabled several important modifications in RRP technique. Walsh and Donker defined the anatomy of the paraprostatic cavernosal nerve bundles and described a method of nerve preservation during retropubic prostatectomy in 1982. This anatomic approach has reduced blood loss, urinary incontinence, and erectile dysfunction, respectively [12]. These advancements, along with other modifications to technique, defined the anatomic RRP which serves as the ‘‘gold standard” of oncologic cure, preservation of continence, and potency by which all treatments of prostate cancer attempt to achieve [7]. RRP has been the mainstay of definitive oncologic treatment for early-stage prostate cancer for decades.

Young first used a perineal approach to do RP in 1904 [13]. Belt modified the Young RPP by approaching the prostate through the perineum between the longitudinal fibers of the rectum and the circular fibers of the external anal sphincter in 1942 [14]. In 1985, Weiss et al. applied this information to develop an NS technique during total perineal prostatectomy [15]. In 1988, Weldon and Tavel demonstrated that nerve sparing (NS) techniques could be applied to the perineal approach [16]. Harris and Thompson modified the technique of RPP to incorporate early dissection of the vas and seminal vesicles, bladder-neck preservation, and NS techniques in the early1990s [17]. In experienced hands, RPP is able to achieve complete cancer resection while preserving urinary and sexual function in the majority of men presenting with clinically localized prostate cancer [18].

**Figure 1:**
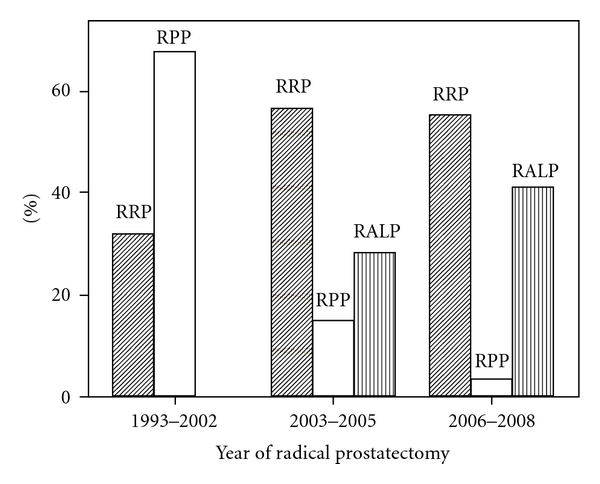
Radical prostatectomies over time at the Duke Prostate Center, Duke University Medical Center.

In the 1990s, laparoscopic prostatectomy techniques were developed [19]. However, due to the technical difficulty of the procedure, this operation failed to attain widespread use until the advent of the da Vinci robotic interface by Intuitive Surgical [20]. RALP has been slowly incorporated into the mainstream of urologic practice since Binder and Kramer applied it in 2000 [21]. Menon et al. standardized the RALP technique by describing the Vattikuti Institute Prostatectomy [22]. Since that time, the use of RALP has steadily increased [23]. RALP is rapidly becoming the predominant form of surgical management for prostate cancer in the United States. In 2007, about 60–70% of all radical prostatectomies in the US were performed by assistance of the “da Vinci” surgical system (personal communication, Intuitive Surgical) [24, 25]. Hu et al. identified 14,727 men undergoing minimally invasive, perineal, and retropubic RP from 2003 to 2005 using nationally representative, employer-based administrative data. Minimally invasive radical prostatectomy use increased from 6.2% in 2003 to 21.6% in 2005, while retropubic and perineal radical prostatectomy use decreased from 86.6% to 72.8% and 5.2% to 4.1%, respectively [2]. At the Duke Prostate Center, RPP was the main operative technique before 2003. In 2003 to 2005, the rates of RRP, RPP, and RALP were 56.7%, 14.9%, and 28.4%, respectively. Compared between 2006–2008 and 2003–2005, RRP decreased from 56.7% to 55.4%, RPP decreased from 14.9% to 3.5%, but RALP increased from 28.4% to 41.1% (Figure 1). Multiple series of RALP are now mature enough to demonstrate safety, efficiency, and reproducibility of the procedure, as well as oncologic and functional outcomes comparable to RRP. Further prospective, randomized studies comparing both surgical techniques will be necessary in order to draw more definitive conclusions [26].

## 3. Nerve Sparing

The technique for anatomic NS radical prostatectomy was reported in 1983 by Walsh et al. [27]. There are two surgical approaches for this procedure. In the so-called “anatomical technique,” reported by Walsh, the nerves dissection is initiated at the apical level with primary isolation of the urethra [28]. Ruckle and Zincke have proposed an alternative technique where the neurovascular bundles are primarily dissected off the lateral prostate and subsequently the urethra [29]. Several variations of this lateral approach to the neurovascular bundles have been described [30, 31]. A majority of authors agree that the ideal candidate for an NS procedure should be fully potent preoperatively and have an organ-confined cancer, that is, a clinical T1/T2a-b disease [32]. As the neurovascular bundles lie outside the capsule and fascia of the prostate, cancer control should not be compromised by an NS procedure when the tumor is organ confined. Cheng et al. found the combination of prostate-specific antigen (PSA) value and the percentage of cancer in the biopsy specimen to predict the risk for positive margins and proposed a model based on these two variables to select the candidates for NS [33]. Park et al. have adopted the practice of sparing the nerve only on the site of a negative biopsy [34]. The utility of using such strict selection criteria is also questioned by the observation that 78% of patients with unilateral positive biopsies have bilateral tumor involvement at the examination of the entire specimen [35]. Walsh et al. state that the site of a positive biopsy, a palpable tumor, or the presence of perineural invasion represents strict criteria upon which the decision to excise a neurovascular bundle should be based [27]. Walsh et al. also recommend a subjective intraoperative judgment as the most accurate indicator of the necessity to sacrifice the neurovascular bundle. Secondary excision of the neurovascular bundles lies in the presence of one of the following intraoperative findings: (1) induration in the lateral pelvic fascia; (2) adherence of the neurovascular bundle to the prostate while it is been released; (3) Inadequate tissue covering the posterolateral surface of the prostate once the prostate has being removed [36].

With the adoption of anatomic RP with cavernous nerve preservation by many surgeons [37], the rate of postoperative recovery of erectile function sufficient for sexual intercourse has improved dramatically from that of the previous era. At major academic centers staffed by highly experienced surgeons, reported rates of erectile function recovery range between 60% and 85% [38, 39]. The recent comparative studies of men undergoing open versus RALP using the same methodology for capturing potency and the same definition of potency failed to show any advantage of the robotic approach [40]. Several investigators have subsequently reported on potency rates based upon NS status (Table 1). There are no differences between the year before and after 2003 on potency rates in unilateral nerve sparing (UNS) and bilateral nerve sparing (BNS). Also, there is no confirmed evidence to show which type of operation is the best in sexual function recovery among RRP, RPP and RALP. Compared to bilateral nerve sparing (BNS), the unilateral excision of neurovascular bundles will compromise potency rates to about 15% to 20% of patients. Brehmer et al. also reported unilateral nerve preservation was performed in 88 of the 92 patients with histologically confirmed unilateral prostate cancer. A proportion of 48% (15/31) of the patients followed for more than 24 months and who had a good erectile function prior to surgery reported unassisted sexual intercourse [41]. Briganti et al. reported the first preoperative risk stratification tool aimed at assessing the probability of erectile function recovery after BNSRP. This study included 435 patients treated with retropubic BNSRP between 2004 and 2008 at a single institution. They stratify patients into three groups according to the risk of erectile dysfunction after surgery: low (age ≤ 65 years, IIEF-EF ≥ 26, Charlson comorbidity index (CCI) ≤ 1; *n* = 184), intermediate (age 66–69 years or IIEF-EF 11–25, CCI ≤ 1; *n* = 115), and high (age ≥ 70 years or IIEF-EF ≤ 10 or CCI ≥ 2; *n* = 136). The 3-year erectile dysfunction recovery rate significantly differed between the three groups, being 85, 59, and 37% in patients with low, intermediate, and high risk of postoperative erectile dysfunction, respectively (*P* < 0.001). Multivariable Cox regression analysis confirmed a highly significant association between the risk classification and erectile dysfunction recovery (*P* < 0.001). The proposed patient stratification tool showed a 69.1% accuracy [42].

Because the neurovascular bundle is in close proximity to the prostatic capsule, sparing the neurovascular bundle has the potential to transect prostate, or cause a positive surgical margin (SM+) in a region of extracapsular disease. The surgeon considering an NS procedure must balance the need for complete eradication of local tumor with the preservation of sexual function [51]. A successful NS technique should combine a high probability of potency recovery with a low SM+ rate, particularly at the apex and the posterolateral prostate. A large multicenter retrospective review of 9035 RPs performed in the last 20 years found the overall SM+ rate falling from 40% between 1982 and 1986 to 10% between 1997 and 2002. However, the SM+ rate in the pT3 disease population slightly increased between 1997 and 2002, implying that the decrease in SM+ rates is most likely due to stage migration rather than major improvement in surgical technique [52]. Sofer et al. reported that NS status was not a predictor of SM+ status or biochemical recurrence following RP, when used on appropriately selected patients [53].

NS procedure nowadays does not seem to have a significant impact on prostate cancer control as the majority of patients electing surgery have clinically organ-confined disease. Future improvement in diagnostic criteria may help to better identify those patients with extracapsular extension for whom the preservation of bundles may translate into SM+ [54].

The technique of NS during RP was described more than 20 years ago. The operation is currently routinely performed worldwide. UNS and BNS do not increase the probability of SM+ or biochemical recurrence after RP when patients are properly selected. Based on literatures, NS is a safe means of preserving potency on well-selected patients undergoing RP [55].

## 4. Surgical Margin

**Figure 2:**
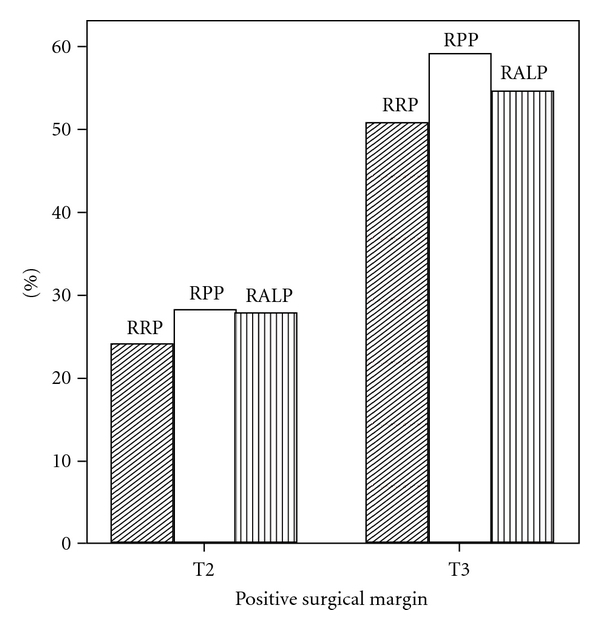
Positive surgical margin rate in pT2-3 diseases (*n* = 2270 between 1993 and 2008, data from Duke Prostate Center).

SM+ is defined as extension of the tumor to the inked surface of the resected specimen [56, 57]. Among patients with organ confined tumors, the 10-year PSA progression-free survival rates are approximately 85%, but are reduced to about 55% among those with an SM+ [58]. Swindle et al. reported 1,389 consecutive patients with clinical stage T1-3 prostate cancer treated with RP from 1983 to 2000. They found that 179 patients (12.9%) had a SM+, including 6.8% of 847 patients with pT2 and 23% of 522 patients with pT3 [59]. In a report of 301 consecutive RPP patients, SM+ rates were reported as 24.7% [60]. Zorn et al. reported the SM+ rates in 300 RALP patients from 2003–2005 were 15.1% for pT2 and 52.1% for pT3 disease [61]. Korman et al. reported a retrospective study of 60 patients who underwent RRP and 40 patients who underwent RPP by the same surgeon. The two groups had comparable clinical stage and Gleason grades. Although, there was no long-term followup, there was no significant difference in the SM+ rate in the retropubic and perineal procedures (16 versus 22%, *P* = 0.53) [62]. Another report about SM+ rate was also not significantly different between RRP and RPP (18.9 versus 13.9%) [63]. The study using a total of 1,747 patients underwent RALP (*n* = 1238) and RRP (*n* = 509) between July 2002 and December 2006 found the incidence of SM+ was 16 of 171 (9.4%) versus 33 of 137 (24.1%) for pT2 and 14 of 28 (50%) versus 36 of 60 (60%) for pT3 disease [64].  Figure 2 shows the Duke Prostate Center SM+ rate for all patients stratified by pathology stage. The SM+ rates at our center were 24.0% in RRP group, 28.2% in RPP group, and 27.3% in RALP group in pT2 disease, and 50.6%, 59.2% and 54.7% in pT3 disease. There were no significant differences among RRP, RPP, and RALP.

There are several reports that have consistently reported that an SM+ represents an independent predictor of biochemical recurrence (BCR) after RP [65–67]. The 5-year actuarial BCR rates are 29.4% reported by Orvieto et al. [68] and 33% reported by Blute et al. [69]. Multiple SM+ have been shown to carry a worse prognosis in virtually all studies. A multivariate analysis on the number of SM+ (solitary versus multiple) showed that there was a statistically significantly greater risk of recurrence in patients with more than one SM+, with a hazard ratio of 2.19 at the 95% CI [52]. The locations of SM+ were recorded for 1308 consecutive men who underwent RP between 2000 and 2006. The BCR rate at 5 years for men with a SM+ was 49.4%. The 5-year actuarial BCR rates were dependent on the site of the SM+ (*P* = 0.035 [70]). 

Hashimoto et al. retrospectively analyzed 238 patients with prostate cancer who underwent RRP and bilateral pelvic lymph node dissection from 1985 to 2005. The 5-year BCR-free survival rates were 81.7% and 62.6% in patients with negative surgical margin (SM−) and SM+, respectively (*P* < 0.001) [71]. Barocas et al. compared BCR-free survival of patients who underwent RRP versus RALP in concurrent series at a single institution. There were 491 RRP (25.9%) and 1,413 RALP (74.1%) performed. The 3-year BCR-free survival rate was similar between RRP and RALP groups on the whole as well as when stratified by margin status. In RRP group, the 3-year BCR-free survival rate was pT2 SM− 96.6% (89.9–98.9%) versus pT2 SM+ 83.1% (66.4–91.9%) and pT3 SM− 69.6% (53.9–80.9%) versus pT3 SM+ 51.6% (35.5–65.6%). In RALP group, the 3-year BCR-free survival rate was pT2 SM− 95.0% (91.1–97.2%) versus pT2 SM+ 83.9% (68.5–92.2%) and pT3 SM− 67.4% (52.0–78.8%) versus pT3 SM+ 42.3% (26.6–57.2%) [72].  Table 2 shows the Duke series in terms of outcomes after RP by SM+ versus SM−. While there is no question that an SM+ has an impact, the magnitude at 10-year followup may be less than many patients perceive. While there is a decrement of almost 10% for 10-year metastases-free survival (85% versus 94%), the difference in cancer-specific survival is less than 7% (89.8% versus 96.5%, [73]).

Although RP is used in patients with the assumption that there is no locally advanced or metastatic spread, SM+ rates are still significant [74]. As seen in previous studies, patients with SM+ are at greater risk of progression. SM+ rates of RP not only affect the recurrence but also the metastases-free survival and cancer-specific survival of prostate cancer.

### 4.1. Anastomosis

One of the critical steps of RP that may influence the rate of postoperative complications is the anastomosis of the bladder to the urethral stump. The general principle to achieve this, beside the anastomotic technique used, is a watertight, tension-free anastomosis with mucosal-to-mucosal coaptation and proper urethral alignment. Historically, the number of six sutures was described by Walsh to be used for the vesicourethral anastomosis [28]. Four, two, even a running suture (9) technique were used in clinical practice [75, 76]. Gallo et al. compared three groups of patients who had undergone RRP. The patients were randomly assigned to undergo six, four and 2 sutures vesicourethral anastomosis techniques. The low number of sutures in the 2-suture vesicourethral anastomosis technique reduces operating times, does not influence perioperative and intraoperative parameters, and results in excellent functional outcome [77]. The traditional reconstruction of the bladder neck describes eversion of the mucosa, which was thought to reduce the incidence of bladder neck contracture [27]. Srougi et al. reported the role of mucosal eversion during reconstruction of the bladder neck in a randomized study of 95 RRP patients. Based on historical data, the authors considered that mucosal eversion in vesicourethral anastomosis may not be necessary and could even be deleterious if it increased the risk of fistula and excessive fibrosis. They found that there was no difference in the rate of urinary leakage, bladder neck constracture, and continence at 1-year followup in patients randomized to bladder neck eversion versus no eversion. In general, RALP does not incorporate mucosal eversion and has demonstrated considerably lower bladder neck constracture rates when compared with open series [78]. Gillitzer et al. reported 866 RPP and 2052 RRP for localized prostate cancer. Median follow-up was 52 months (12–136). The rate of anastomotic bladder neck stricture after RPP and RRP was 3.8% (33/863) and 5.5% (113/2048), respectively (*P* = 0.067, [79]). Msezane et al. reviewed the literatures of RRP and RALP and revealed an incidence of bladder neck contracture ranging from 1% to 17.5% in RRP and 0.6% to 4.1 in RALP from many of the large series. Variations of bladder mucosal eversion and anastomotic suturing (interrupted versus continuous) were noteworthy. Similarly, the length of Foley catheterization is significantly longer in RRP series (10–14 days) when compared with RALP series (4–7 days) [80].

## 5. Complications of RP

The comparison of perioperative complications is also an important method to assess the advancements of operative techniques. The study of Lance et al. shows the relatively low morbidity of both RPP and RRP approaches. RPP had an advantage of lower estimated blood loss and homologous transfusion rates but a higher rectal injury rate than RRP [81]. Between January 2002 and August 2007, a series of 1738 consecutive patients underwent RALP (*n* = 1253) and RRP (*n* = 485) for clinically localized prostate. Overall, 170 patients required blood transfusions (9.7%), and were 112 patients (23%) in RRP group compared with 58 patients (4.8%) in the RALP group. Infectious complications occurred in 44 RRP patients (9%) compared with 18 (1%) in the RALP group. Bladder neck contracture was treated in 22 (4.5%) patients who had undergone RRP compared with 3 (0.2%) in the RALP group. The RALP has resulted in a decrease in the number of patients who require blood transfusions and decreased numbers of patients with postoperative wound infections [82]. Krambeck et al. assessed the perioperative complications in a comparative study matching RRP and RALP groups. There was no significant difference in overall perioperative complications between the RALP and RRP groups (8.0 versus 4.8%, *P* = 0.064, [48]).  Table 3 shows that RPP has more rectum injuries before 2000 years. In the era of 2000–2004, there is no significant difference between RRP and RPP in perioperative complications. RALP is becoming a common technique in treating prostate cancer after 2004. The RALP has less blood loss and blood transfusion than open RP in most series. 

## 6. Impact on Urinary and Sexual Functions

Urinary incontinence can be a devastating complication following prostatectomy. Given the difficulty in measuring this clinical outcome, however, continence results in the literature vary widely. Most centers report continence rates between 84% and 96% (Table 4). Continence rates improve with follow-up time after RP. Matsubara et al. in 2005 evaluated the impact of RPP on urinary continence and quality of life. Urinary function returned to the preoperative baseline level by 6 months postoperatively. The majority of patients who underwent RPP rapidly regained urinary continence and quality of life within 3–6 months [92]. Zuo and Hiraoka reported clinical comparative evaluation of RRP and RPP approaches for prostate cancer. There were no differences between the RRP and RPP groups in incontinence rates [93]. Boris et al. compared perioperative, functional, and oncological outcomes of a single surgeon's experience with RRP, RPP, and RALP. Urinary continence (one pad or less) at 12 months was 96% in RRP, 96% in RPP, and 96% in RALP group, no major differences in urinary continence among the RRP, RPP, and RALP groups [94]. A nonrandomized prospective comparative study with patients undergoing RALP or RRP for clinically localized prostate cancer from February 2006 to April 2007 showed that the 12-month continence rates were 88% after RRP and 97% after RALP (*P* = 0.01, [95]).

Before the development of an anatomic approach to RP, virtually all patients developed erectile dysfunction following RP. The realization that erectile dysfunction arose from damage to an anatomically distinct network of autonomic nerves to the corpora cavernosa led to modifications in surgical technique, with vastly improved potency outcomes [12]. The cohort study of the Cancer of the Prostate Strategic Urologic Research Endeavor, comprising 29 academic and community-based sites across the United States, established a 75% potency rate after RP among men aged less than 65 years [100]. Although anatomic NS radical prostatectomy might be performed with expert precision, promising a high likelihood of postoperative recovery of erectile function, many men will nonetheless require as much as 1 year or longer to recover satisfactory functional status. The relative merit of either approach for RP in preventing erectile dysfunction remains a subject of debate (Table 5). 

In most series, a correlation is found between the number of spared neurovascular bundles and the recovery of potency. Kundu et al. reported on 1843 patients operated between 1983 and 2003. The potency rate was 78% in men who have had a BNS procedure and 53% after a unilateral nerve sparing RP [39]. Noldus et al. reported less favourable outcomes. They performed a study on 289 patients operated between 1992 and 1999 and found potency rates of 51.7% and 16.1% after bilateral and unilateral NS procedures, respectively. NS technique also improves the outcomes of urinary continence and potency [103]. Burkhard et al. prospectively assessed the role of NS surgery on urinary continence of RRP. The incidence of incontinence after open RRP is low, and continence is highly associated with an NS technique [101]. Therefore, NS should be attempted in all patients if the principles of oncological surgery are not compromised. 

Melman et al. in 2004 concluded that the anatomic RPP preserves urinary and sexual function as well as RRP [104]. Martis et al. reported that a randomized study of 200 patients underwent an RP performed by retropubic (100 patients) or perineal (100 patients) approach between 1997 and 2004. Differences between SM+ and urinary continence in the two groups were not statistically significant at 6 and 24 months. Differences between erectile function at 24 months were statistically significant in favor of RRP [102]. Parsons et al. compared outcomes of RRP and RALP using evidence-based analysis. There were also no significant differences in 1-year urinary continence (*P* = 0.49) and 1-year erectile function (*P* = 0.09, [105]). So it is difficult based on the current literature to determine if one approach is superior to the other in the outcomes of urinary and sexual function.

## 7. Combined Use of RP and Other Treatment Options

In intermediate- and high-risk localized prostate cancer, several randomized trials have attempted to quantify potential benefits of hormone therapy prior to radical prostatectomy. Most of these studies used a 3-month neoadjuvant treatment period before surgery, and most of them used complete androgen blockade (CAB). A decrease in the postoperative SM+ is consistently seen. However, a benefit in overall survival (OS) has not been observed in any trial. A total of 402 patients were randomized between CAB for 3 months followed by RP or RP alone. A significant difference in pathologic downstaging (15% versus 7%), percentage with SM+ (27% versus 46%), and local relapse rates for cT2 patients (3% versus 11%) was observed, favoring the neoadjuvant group. There were no difference in OS rates, with 93% and 95% of patients alive in the treatment and control groups, respectively (*P* = 0.64, [106]). Similarly, Klotz et al. reported a 6-year follow-up period and also reported no difference in OS (*P* = 0.38), with 5-year OS rates of 88.4 and 93.9%, respectively [107]. There are also studies analyzing disease-free survival. In the study with the longest follow-up, Aus et al. showed no significant difference in PSA progression-free survival rates (49.8% for the neoadjuvant treatment and 51.5% for the prostatectomy treatment, *P* = 0.588, [108]). Soloway et al. recruited patients with clinical stage T2bNxM0 and also reported that there was no difference in the BCR rate. PSA was less than 0.4 ng/ml in 64.8% of the patients in the neoadjuvant androgen ablation plus prostatectomy and 67.6% in the prostatectomy only group (*P* = 0.663, [109]). A meta-analysis published in 2000 analyzed the routine use of neoadjuvant HT before RP. Six of the 7 studies reviewed noted a decrease in the SM+ rate, but no significant improvement in survival was observed [110]. Neoadjuvant androgen-deprivation therapy (ADT) affects the rate of SM+; benefits in survival have not been shown. As such, the routine use of neoadjuvant hormonal therapy (HT) before definitive RP is not recommended outside a clinical trial [111]. Hormone therapy combined with prostatectomy is associated with significant clinical benefits in patients with local or locally advanced prostate cancer. Significant local control may be achieved when given prior to prostatectomy, which may improve patient's quality of life. When given adjuvant to these primary therapies, hormone therapy not only provides a method for local control, but there is also evidence for a significant survival advantage [112]. 

Because high-risk patients with prostate cancer can be readily identified by clinical criteria, many studies have attempted to use local and systemic adjuvant therapy to reduce the risk of recurrence and improve survival. In a study of node-positive disease after RP, Messing et al. reported that at a median followup of 7.1 years, OS was better in the immediate HT group compared to the control group (7 out of 47 deaths compared to 18 of 51 in the observation group, *P* = 0.02) and a significant improvement in disease-free survival with adjuvant HT (*P* < 0.001, [113]). In 2005, McLeod et al. reported 4454 patients on bicalutamide who underwent RP and reported an improvement in progression-free survival in RP group after a median of 7.4 years (HR 0.75, 95% CI 0.61–0.91, *P* = 0.004). There was no improvement in OS in both the local (HR 1.0, 95% CI 0.80–1.26) or the locally advanced (HR 1.09, 95% CI 0.85–1.39, *P* = 0.51) subgroups [114]. Siddiqui et al. reported a retrospective series from the Mayo Clinic with patients who underwent RP and had negative lymph nodes. A total of 580 patients were treated with adjuvant ADT) and 1160 were observed only. A significant benefit in 10-year biochemical progression-free survival (95% versus 90%) and cancer-specific survival (98% versus 95%) was noted, favoring ADT therapy. However, no significant difference was observed in OS (83% for ADT and observation groups, [115]). Although the treatment is still controversial, most specialists in genitourinary cancers advocate the use of adjuvant ADT in patients who have undergone RP with lymph node-positive disease. The exact duration of therapy is debated. In patients without lymph node-positive disease, there remains no conclusive evidence for the use of adjuvant HT after definitive therapy with RP [111]. 

Adjuvant radiotherapy (RT) after RP has been shown in recent large randomized trials to increase disease-specific death-free rate and OS compared with no adjuvant RT. The recent European Organization for Research and Treatment of Cancer (EORTC) 22911 study recruited patients with high-risk factors (including SM+, capsule invasion, and seminal vesicle invasion) and randomized them to immediate postoperative RT (median dose 60 Gy over 6 weeks) or a watchful-waiting policy. Both the local control rate and BCR rate improved after immediate treatment. The biochemical progression-free survival rate at 5 years was 72.2% in the adjuvant arm, compared to 51.8% in the watchful-waiting group; the clinical progression-free survival at 5 years was 83.3% and 74.8%, respectively. Local or regional failure was also significantly lower in the immediate adjuvant treatment. All of these results were statistically significant [116]. An update of this trial suggested that the benefit was restricted to the patients with SM+ (HR 0.38, 95% CI 0.26–0.54), compared with patients with SM− (HR 0.88, 95% CI 0.53–1.46, [117]). Another trial randomized 425 patients with pT3N0 prostate cancer to adjuvant RT (60–64 Gy) or usual care plus observation. After a long median follow-up, the adjuvant RT group had a significant increase in OS (15.2 versus 13.3 years; HR 0.72, 95% CI 0.55–0.96) and metastasis-free survival (14.7 versus 12.9 years; HR 0.71, 95% CI 0.54–0.94). Subset analyses have shown that this benefit was independent of the Gleason score and included patients with or without detectable PSA postprostatectomy and those with positive margins or seminal vesicle involvement [118]. From previous data, we can conclude that subsets of patients with pathologically advanced prostate cancer benefit from RT adjuvant therapy. However, whether or not true adjuvant therapy is superior to salvage radiotherapy for early PSA recurrence remains hotly debated.

## 8. New Techniques in RP Surgery and RP Future

Open RP is the standard treatment for localized prostate cancer. However, the procedure has inherent morbidity associated to it. Therefore, less invasive surgical techniques have been sought; one such alternative is RALP. The advantages provided by robotic technology have the potential to minimize patient morbidity while improving both functional and oncological outcomes. Although it is a recent technological advancement, robotic surgery has shown an increasing rate of adoption worldwide. Currently, more than 30,000 patients have undergone this procedure worldwide [1]. Recent literatures have compared RRP and RALP. The blood loss is greater in RRP; the hospital stay is shorter for RALP than for RRP. There was no significant difference in SM+, urinary continence, and erectile function among RRP and RALP [105, 119–121]. The RALP procedure has prompted many expert RRP surgeons to further modify techniques (smaller incision, local anesthesia infiltration into the incision, intraoperative normovolumic hemodilution to avoid transfusion, and better care pathways) to lessen differences [122]. 

Imaging of the prostate is suboptimal. While transrectal ultrasound can help image the gland to direct diagnostic needle biopsy, the current ultrasound platform cannot accurately image intraprostatic pathology. Newer imaging methods have been developed, including contrast-enhanced color Doppler ultrasound, elastography, dynamic contrast-enhanced magnetic resonance imaging (DCE-MRI), and magnetic resonance spectroscopic imaging (MRSI). Contrast-enhanced color Doppler ultrasound targeted biopsies have shown that the targeted approach detects more cancers and cancers with higher Gleason scores with a reduced number of biopsy cores and reduce the cost and morbidity associated with the diagnosis of prostate cancer [123]. Elastography for the assessment of tissue elasticity has been demonstrated to be useful for the detection of prostate cancer and may further improve prostate cancer staging [124]. In clinical practice, the fusion of MRI or DCE-MRI with MR MRSI may improve the evaluation of cancer location, size, and extent, while providing an indication of tumor aggressiveness. Pretreatment knowledge of these prognostic variables is essential for achieving minimally invasive, patient-specific therapy [115, 125–127].

## 9. Conclusions

The clinical incidence of prostate cancer continues to increase in the patient population, and urologists struggle to identify those patients who require intervention for their disease and to determine which treatment modality is best. Active surveillance, brachytherapy, external beam radiation therapy (EBRT), HT, and RP are the current options for prostate cancer treatment, each with a distinct impact on a patient's health-related quality of life. For many patients with a long life expectancy, RP remains the most effective approach with respect to both oncologic success and maximization of quality of life [1, 128–130]. 

The perineal route was the first to be developed, but it has fallen out of favor due to the need of performing obturator lymphadenectomy by a separate approach. RPP is still a reasonable approach for obese patients, patients with prior pelvic surgery, or patients with prior pelvic radiation [131]. RALP has become popular among surgeons because of ease of pelvic access, high-power magnification, minimal bleeding, and decreased blood transfusions during the operation [132]. However, it has not yet become the firmly established standard of care because long-term outcomes have yet to be established. By contrast, long-term data have confirmed the efficacy of RRP with disease control rates of 60% to 75% at 10 years and cancer-specific survival rates of 97% and 95% at 10 and 15 years, respectively [133]. To date, there is no reason that a surgeon obtaining excellent functional and oncologic results with RRP should change to a different approach. Due to the limitations of the currently available literature, further prospective, randomized comparative studies are needed [6].

## Figures and Tables

**Table 1:** Effect of nerve sparing in radical prostatectomy on sexual function recovery.

Author	Type	No. of patients	Followup months	Potent (%)		*P*
				UNS	BNS	
Before and up to 2003						
Quinlan et al. (1991) [43]	RRP	503	18	56	76	NA
Weldon et al. (1997) [44]	RPP	50	18	68	73	NA
Catalona et al. (1999) [45]	RRP	858	18	47	68	NA
Stanford et al. (2000) [46]	RRP	938	>18	41	44	NA
After 2003						
Kundu et al. (2004) [39]	RRP	1834	18	53	76	NA
Harris (2007) [18]	RPP	140	12	27	49	0.02
Marien and Lepor (2008) [47]	RRP	1110	24	44	60	0.011
Krambeck et al. (2009) [48]	RRP	807	>6	53	72	<0.001
Menon et al. (2007) [49]	RALP	1142	12	58	70	NA
Hakimi et al. (2009) [50]	RALP	60	12	64	77	NA

UNS: Unilateral nerve sparing; BNS: Bilateral nerve sparing; RRP: Radical retropubic prostatectomy; RPP: Radical perineal prostatectomy; RALP: Robotic-assisted laparoscopic prostatectomy; NA: Not applicable.

**Table 2:** Outcomes after radical prostatectomy and impact of positive and negative surgical margins [72].

	SM+ (% ± SE)	SM− (% ± SE)	*P*
PSA recurrence-free survival			0.0001
1 year	69.2 ± 1.5	82.2 ± 0.8	
2 years	61.9 ± 1.6	76.6 ± 0.9	
5 years	51.3 ± 1.8	67.9 ± 1.0	
10 years	43.2 ± 2.6	61.8 ± 1.2	
Distant metastasis-free survival			0.0001
1 year	98.9 ± 0.3	99.4 ± 0.2	
2 years	97.3 ± 0.6	99.1 ± 0.2	
5 years	93.6 ± 0.9	96.9 ± 0.4	
10 years	85.3 ± 1.9	94.0 ± 0.7	
Disease-specific death-free survival			0.0001
1 year	99.8 ± 0.2	99.8 ± 0.1	
2 years	99.3 ± 0.3	99.6 ± 0.1	
5 years	97.1 ± 0.7	98.7 ± 0.3	
10 years	89.8 ± 1.7	96.5 ± 0.5	

Data from Duke Prostate Center, *n* = 3605.

**Table 3:** Perioperative complications in different types of radical prostatectomies.

Author	Type	No. of patients	Blood loss	Blood transfusion (%)	Rectum (%)	Ureter (%)	Lymphocele (%)	Infection (%)
<2000								
Dillioglugil et al. (1997) [83]	RRP	472	NA	NA	0.6	0.2	2.2	7.3
Mokulis and Thompson (1997) [84]	RPP	60	NA	8.3	10	NA	0	1.7
Catalona et al. (1999) [45]	RRP	1870	NA	NA	0.05	NA	1	0.8
2000–2004								
Lance et al. (2001) [81]	RPP	190	802	15.8	4.9	NA	NA	NA
	RRP	190	1575	10.5	0	NA	NA	NA
Lau et al. (2001) [85]	RRP	1000	NA	NA	0.5	0.1	0.1	NA
Salomon et al. (2002) [86]	RPP	119	NA	15.9	0.8	0.8	1.7	NA
Augustin et al. (2003) [87]	RRP	1243	NA	29	0.4	0.3	2.9	0.7
>2004								
Ghavamian et al. (2006) [88]	RRP	70	563	31.4	NA	NA	2.8	4.1
Hu et al. (2006) [89]	RALP	322	NA	1.6	0.6	0.3	0.9	1.9
Ahlering et al. (2006) [90]	RALP	1130	NA	0.3	0.5	NA	NA	0.2
Fischer et al. (2008) [91]	RALP	210	100–300	1	0.5	0	5	5

RRP: Radical retropubic prostatectomy; RPP: Radical perineal prostatectomy; RALP: Robotic-assisted laparoscopic prostatectomy; NA: Not applicable.

**Table 4:** Recovery of continence rates following RRP, RPP, and RALP.

Author	Type	No. of patients	Criteria	Urinary continence (%)		
				3 months	12 months	24 months
Eastham et al. (1996) [96]	RRP	581	No pad	65	92	95
Harris and Iselin (2003) [97]	RPP	508	No pad	62	96	NA
Roumeguere et al. (2003) [98]	RRP	51	No pad	62.5	83.9	NA
Lepor et al. (2004) [99]	RRP	621	No pad	74.4	92.4	97.1
Matsubara et al. (2005) [92]	RPP	41	No pad	65	87	NA
Menon et al. (2007) [49]	RALP	1142	No pad	NA	84	NA
Zorn et al. (2007) [61]	RALP	161	No pad	47	90	92
Krambeck et al. (2009) [48]	RRP	564	No pad	NA	93.7	NA
		286	No pad	NA	91.8	NA

RRP: Radical retropubic prostatectomy; RPP: Radical perineal prostatectomy; RALP: Robotic assisted laparoscopic prostatectomy; NA: Not applicable.

**Table 5:** Recovery of erectile function rates following RRP, RPP, and RALP.

Author	Type	no. of patients	Criteria	Erectile function (%)		
				3 months	12 months	24 months
Weldon et al. (1997) [44]	RPP	220	Intercourse	NA	50	70
Roumeguere et al. (2003) [98]	RRP	51	Intercourse	33.3	54.5	NA
Graefen et al. (2006) [101]	RRP	1755	Intercourse	NA	56	NA
Martis et al. (2007) [102]	RRP	100	Intercourse	NA	60	NA
	RPP	100	Intercourse	NA	40	NA
Zorn et al. (2007) [61]	RALP	161	Intercourse	53	80	82

RRP: Radical retropubic prostatectomy; RPP: Radical perineal prostatectomy; RALP: Robotic-assisted laparoscopic prostatectomy; NA: Not applicable.

## Abbreviations

ADT: Androgen-deprivation therapy
BCR: Biochemical recurrence
BNS: Bilateral nerve sparing
CAB: Combined androgen blockade
DCE-MRI: Dynamic contrast-enhanced magnetic resonance imaging
EBRT: External beam radiotherapy
HIFU: High-intensity focused ultrasound
HT: Hormonal therapy
LRP: Laparoscopic radical prostatectomy
MRSI: Magnetic resonance spectroscopic imaging
NA: Not applicable
NS: Nerve sparing
OS: Overall survival
PSA: Prostate-specific antigen
RALP: Robotic-assisted laparoscopic radical prostatectomy
RP: Radical prostatectomy
RPP: Radical perineal prostatectomy
RRP: Radical retropubic prostatectomy
RT: Radiotherapy
SM+: Positive surgical margin
UNS: Unilateral nerve sparing
VTP: Vascular-targeted photodynamic therapy.
